# Noncanonical Expression of a Murine Cytomegalovirus Early Protein CD8 T-Cell Epitope as an Immediate Early Epitope Based on Transcription from an Upstream Gene

**DOI:** 10.3390/v6020808

**Published:** 2014-02-14

**Authors:** Annette Fink, Julia K. Büttner, Doris Thomas, Rafaela Holtappels, Matthias J. Reddehase, Niels A. W. Lemmermann

**Affiliations:** Institute for Virology and Research Center for Immunology (FZI), University Medical Center of the Johannes Gutenberg-University Mainz, Obere Zahlbacher Str. 67, Mainz D-55131, Germany; E-Mails: finka@uni-mainz.de (A.F.); julia_buettner@uni-mainz.de (J.K.B.); thomdo00@uni-mainz.de (D.T.); R.Holtappels@uni-mainz.de (R.H.)

**Keywords:** antigenic peptides, antigen presentation, CD8 T cell epitope, gene expression, immediate-early protein, murine CMV ORF *m164*, open reading frame, RACE mapping, reverse immunology, transcription start site, translation start site

## Abstract

Viral CD8 T-cell epitopes, represented by viral peptides bound to major histocompatibility complex class-I (MHC-I) glycoproteins, are often identified by “reverse immunology”, a strategy not requiring biochemical and structural knowledge of the actual viral protein from which they are derived by antigen processing. Instead, bioinformatic algorithms predicting the probability of *C*-terminal cleavage in the proteasome, as well as binding affinity to the presenting MHC-I molecules, are applied to amino acid sequences deduced from predicted open reading frames (ORFs) based on the genomic sequence. If the protein corresponding to an antigenic ORF is known, it is usually inferred that the kinetic class of the protein also defines the phase in the viral replicative cycle during which the respective antigenic peptide is presented for recognition by CD8 T cells. We have previously identified a nonapeptide from the predicted ORF*m164* of murine cytomegalovirus that is presented by the MHC-I allomorph H-2 D^d^ and that is immunodominant in BALB/c (H-2^d^ haplotype) mice. Surprisingly, although the ORF*m164* protein gp36.5 is expressed as an Early (E) phase protein, the m164 epitope is presented already during the Immediate Early (IE) phase, based on the expression of an upstream mRNA starting within ORF*m167* and encompassing ORF*m164*.

## 1. Introduction

In listings of antigenic peptides identified in viral open reading frames (ORFs) by “reverse immunology” [1,2,3], annotated viral proteins are usually co-listed under the implicit inference that the antigenic peptides are derived by antigen processing when the respective proteins are synthesized, and, thus, belong to the same kinetic class in the cascade regulation of viral gene expression. Although such a “canonical” expression and presentation of an antigenic peptide as a peptide-MHC (pMHC) complex almost certainly applies, because, as far as we are aware of, no viral protein or even self protein is exempt from becoming degraded in one of the antigen processing pathways, the kinetic class assignment of an antigenic peptide and its corresponding protein can nonetheless differ due to “noncanonical” entry into a processing pathway. One mechanism of noncanonical antigen deployment and pre-immediate early presentation is delivery of virion structural proteins during the viral entry process [4,5,6], whereas canonical expression and presentation would occur not before the late (L) phase, close to the time of virion assembly and release and, thus, possibly too late for antiviral immune protection. A second virion entry-associated mechanism could be the delivery of viral RNA enclosed in virions [7], leading to pre-immediate early gene expression independent from the canonical kinetic class of the respective viral RNA. Such mechanisms can be of functional relevance for cell surface presentation of antigenic peptides prior to the expression of viral immune evasion proteins that would otherwise interfere [8,9,10]. Here, we report on an example for a third mechanism of noncanonical antigenic peptide presentation.

We have previously identified an antigenic peptide in ORF*m164* of murine cytomegalovirus (mCMV) by employing the strategy of “reverse immunology” [11]. CD8 T cells derived from infected BALB/c (haplotype H-2^d^) mice were found to respond to MHC (H-2 D^d^) gene-transfected L cells (fibroblasts of the unrelated haplotype H-2^k^) exogenously loaded with high performance liquid chromatography (HPLC)-separated naturally processed peptides derived from mCMV-infected BALB/c mouse embryo fibroblasts (MEF). This was the first indication of the existence of mCMV peptide(s) presented by MHC-I D^d^, as the only antigenic mCMV peptide known at that time was the Immediate Early (IE) peptide IE1 presented by MHC-I L^d^ [12]. Algorithms predicting H-2 D^d^ binding nonapeptides were applied to the viral genome-wide coding sequence and predicted a panel of high-scoring peptides of which a peptide with the amino acid sequence AGPPRYSRI encoded by ORF*m164* was functionally verified by the response of CD8 T cells. Along with the IE1 epitope and with more recently identified epitopes from ORFs *M105* and *m145* [13], the m164 epitope proved to be immunodominant with respect to the frequency of CD8 T cells responding to it during the immune response to acute infection. In addition to the IE1 epitope, which is the prototype of an epitope that induces expansion of the memory CD8 T-cell pool at extralymphoid sites of latent mCMV infection [14], a phenomenon known today as “memory inflation” ([15], reviewed in [16,17,18]), the m164 epitope turned out to be the second inducer of memory inflation in the H-2^d^ haplotype, a property that it shares with three epitopes in the H-2^b^ haplotype [19] and that is thought to indicate episodes of limited viral gene expression during latency (for recent reviews, see [20,21]). As the ORF*m164* protein, a non-essential ER-resident type-I glycoprotein of 36.5 kDa, is expressed as an Early (E) phase protein [22], it was assumed that the m164 peptide is presented not until the E phase. Interestingly, cytolytic T lymphocytes (CD8^+^ CTL) of an m164-specific CTL line (m164-CTLL) recognized infected fibroblasts in the E phase despite all immune evasion molecules being expressed, a finding that was explained by high numbers of pMHC-I (m164 peptide-D^d^) complexes exhausting the inhibitory capacity of the immune evasion proteins, thus allowing the escape of some of the pMHC-I complexes to the cell surface sufficient for recognition [23]. Importantly, in an experimental approach of CMV immunotherapy (for reviews, see [24,25]), m164-CTLL proved to protect against multiple organ CMV disease upon adoptive cell transfer into infected, immunocompromised recipient mice [11].

Here, we report the astounding finding that this “E phase peptide”, as well as a transgenic peptide replacing it in a recombinant mCMV, are recognized by CTLL of the respective cognate specificities on infected cells metabolically arrested in the IE phase, thus representing a case of “noncanonical presentation”. This presentation is not explained by virion entry-dependent delivery of antigenic virion protein or epitope-encoding virion-associated RNA, but is based on IE phase expression of an unrelated mRNA originating in ORF*m167* and encompassing the epitope-encoding sequence in ORF*m164*. These data call for caution in assigning epitope presentation to a kinetic phase based just on the kinetic class to which the corresponding viral protein belongs.

## 2. Results and Discussion

### 2.1. Antigenic Sequences Encoded by the Predicted ORFm164 Are Presented on Infected Cells Metabolically Arrested in the IE Phase as Well as in the E Phase

**Figure 1:**
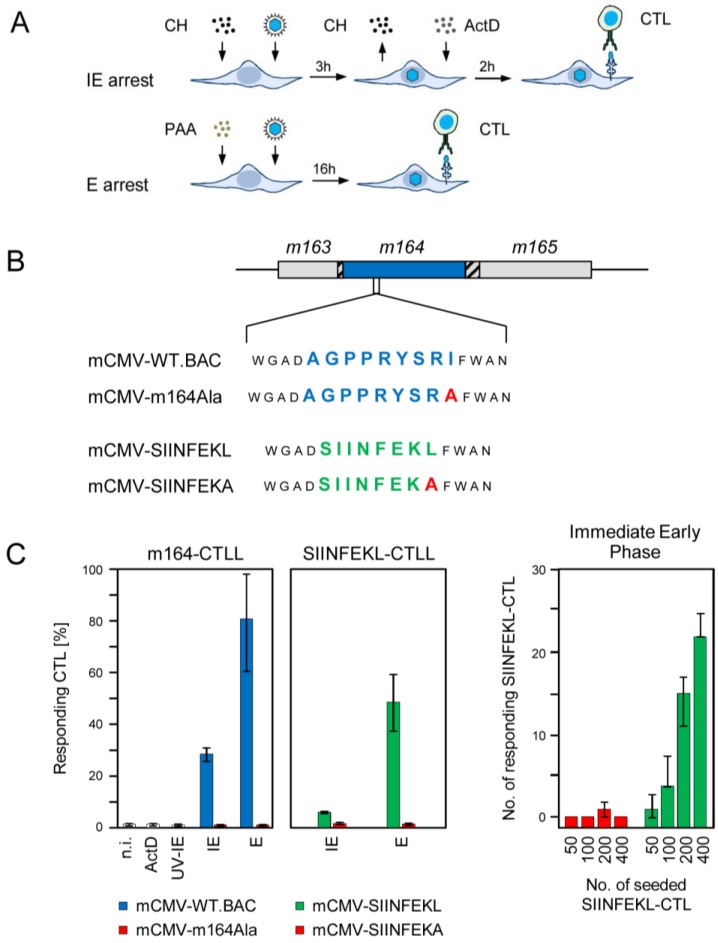
Presentation of intrinsic and transgenic m164 peptides in the IE and E phase. (**A**) Scheme of the experimental protocol for selective arrest of viral gene expression in infected fibroblasts by metabolic inhibitors. For arrest in the IE phase, MEF are infected under conditions of centrifugal enhancement, resulting in an MOI of 4, in the presence of cycloheximide (CH, 100 µg/mL) that becomes replaced at 3 h post-infection (p.i.) with actinomycin D (ActD, 5 µg/mL). For arrest in the E phase, MEF are infected likewise for 16 h in the continuous presence of PAA (250 µg/mL). At 5 h and 16 h p.i., respectively, these cells are used in an IFN-γ-based ELISpot assay as stimulator cells for CTL from CTL lines (CTLL) of defined antigenic peptide specificities; (**B**) Scheme of the mutagenesis rationale for modification of m164 protein antigenicity and immunogenicity in recombinant viruses. Shown is an overview map of the mCMV genomic region encompassing ORFs *m163*-*m165*. ORFs are symbolized by boxes, with the blue box representing ORF*m164* encoding protein gp36.5 that comprises the intrinsic antigenic peptide AGPPRYSRI, which is presented by the MHC-I molecule H-2 D^d^. Hatched boxes indicate overlaps with neighboring ORFs. Amino acid sequences of the intrinsic and the replacing transgenic CD8 T-cell epitope, which is presented by H-2 K^b^, are shown in blue and green one-letter code, respectively. The *C*-terminal residue mutation to Ala is highlighted by red capital letter. Epitope-flanking residues are shown in smaller font; (**C**) Frequencies of cells of the indicated CTLL that respond in an IFN-γ-based ELISpot assay to sensitization by MEF of the presenting MHC haplotype infected with the indicated viruses under the indicated conditions of gene expression phase arrest. (Left two panels) Bars represent most probable numbers determined by intercept-free linear regression analysis based on triplicate assay cultures and graded numbers of CTL seeded; error bars indicate the 95% confidence intervals. n.i., no infection (uninfected MEF); ActD, MEF infected for 5 h in the presence of ActD preventing transcription from the very beginning; UV-IE, IE conditions applied to cells mock-infected with virus inactivated by UV-light (254 nm, *ca.* 4500 J/m^2^). IE and E, MEF infection under conditions of IE and E phase arrest, respectively, as explained above. (Right panel) Bars represent median values of absolute spot counts from triplicate assay cultures for CTL seeded in graded numbers as indicated. Variance bars show the range.

MEFs were infected under established conditions of IE phase or E phase arrest (Figure 1A). For IE phase arrest, infection was performed in the presence of the reversible protein synthesis inhibitor cycloheximide (CH) that becomes replaced after 3 h with the irreversible transcription inhibitor actinomycin D (ActD). This allows for selective and enhanced transcription from IE genes followed by IE protein synthesis, and prevents transactivation of E gene transcription. E phase arrest is achieved by 16 h of infection in the continuous presence of the DNA synthesis inhibitor phosphonoacetic acid (PAA) preventing, by definition, the transition from the E phase to the L phase. The mutation strategy for manipulating antigenicity and immunogenicity of ORF*m164* (reviewed in [26]) is sketched in Figure 1B. In a first approach, MEF were infected either with BAC-cloned wildtype (WT) virus mCMV-WT.BAC coding for the authentic ORF*m164* epitope AGPPRYSRI or with a mutant virus mCMV-m164Ala, in which the *C*-terminal amino acid isoleucine of the antigenic sequence is genetically replaced with alanine. This strategy prevents *C*-terminal proteasomal cleavage of the antigenic peptide in the first place. In addition, it reduces binding affinity to the presenting MHC-I molecule D^d^ in case that inefficient, residual proteasomal cleavage might still generate some AGPPRYSRA peptide. We have previously introduced *C*-terminal residue mutagenesis as an ideal control for testing epitope specificity of the CD8 T cell response [13,27,28,29,30], leaving other parameters of infection *bona fide* untouched. In a second approach, referred to as “orthotopic peptide swap”, the authentic antigenic peptide AGPPRYSRI was replaced at precisely the same site with the ovalbumin-derived model peptide sequence SIINFEKL in recombinant virus mCMV-SIINFEKL [29]. This peptide swap is associated with a switch in the presenting MHC-I molecule, namely from H-2 D^d^ to K^b^. For serving as an epitope specificity control, recombinant virus mCMV-SIINFEKA was generated based on the rationale already explained above [29].

In the experiment to the first approach (Figure 1C, far left panel) m164-CTLL recognized WT virus-infected target cells in the E phase, as it was to be expected. Surprisingly, however, a significant proportion of the m164-CTL, supposedly those with the highest functional avidity [25,31], also recognized target cells arrested in the IE phase. Recognition in both the IE and the E phase was abolished after infection with the epitope deletion virus mCMV-m164Ala, thereby excluding the theoretical possibility that inhibitor treatment of infected target cells might have induced T cell receptor (TCR)-independent, non-epitope specific signaling that activates CTLL. Known noncanonical modes of epitope presentation were also ruled out: (i) infection in the presence of inhibitor ActD prevented presentation of the m164 epitope. This revealed the requirement for *de novo* transcription and thus excluded a noticeable contribution of virion-associated RNA, and (ii) UV-inactivated virus did not lead to the presentation of the m164 epitope, which was not unexpected in light of the fact that no ORF*m164*-encoded protein was detected in the virion proteome [32]. These findings were corroborated by the experiment to the second approach (Figure 1C, center and far right panels). Although the response of SIINFEKL-specific CTL to the K^b^-presented epitope was lower compared to recognition of the authentic m164 peptide by m164-specific CTL, which is not unusual as processing rates and MHC-I binding affinity differ between different epitopes, the key message of presentation in both the IE and E phase was reproduced, and epitope-specificity was confirmed for both phases by missing recognition of cells infected with mCMV-SIINFEKA.

In conclusion, antigenic peptides naturally present or experimentally placed in the sequence of the E phase protein m164/gp36.5 are noncanonically expressed and presented in the IE phase, an obvious paradox that needed to be resolved.

### 2.2. Detection of Distinct mRNA Species in the IE and E Phases by m164 Sequence-Specific Hybridization

**Figure 2:**
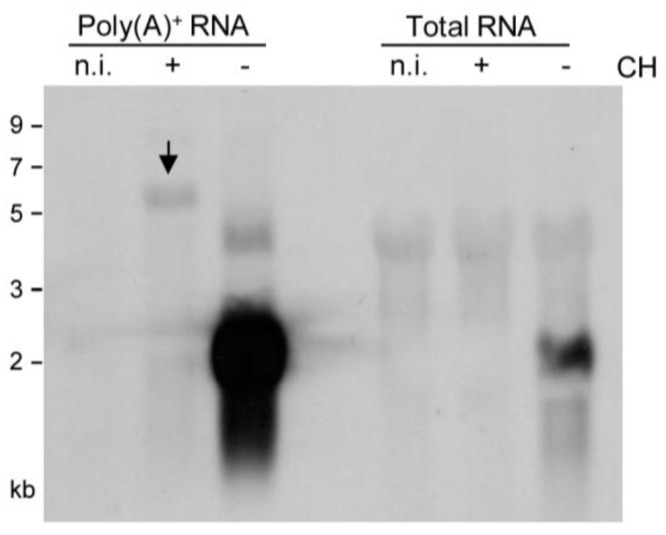
Identification of m164 transcripts by Northern blot analysis. Total cellular RNA or poly(A)^+^ RNA were isolated at 3 h p.i. from MEF infected in the presence (+) or absence (–) of CH (100 µg/mL) and were analyzed by Northern blotting using a DIG-labeled m164-specific RNA probe. The arrow highlights a specific band of >5 kb representing an IE mRNA.

As virion proteins and virion-associated RNA, apparently, did not account for presentation of m164 peptide in the IE phase (recall Figure 1C, left panel), and as recognition by CD8 T cells was clearly epitope-specific (recall Figure 1C, all panels), there must exist an IE mRNA encoding a protein that encompasses the antigenic sequence. Northern blot analysis (Figure 2), using a DIG-labeled m164-specific single-strand RNA-probe for hybridization with total RNA, revealed only an E phase RNA of slightly more than 2 kb in size present 3 h after infection in the absence of protein synthesis inhibition by CH but absent in the presence of CH. When sensitivity was increased by using poly(A)^+^ RNA for hybridization with the riboprobe, this mRNA species was far more prominent but remained absent in cells infected in the presence of CH, thus identifying it as a true E phase transcript. The nature of a low-abundance CH-sensitive E phase transcript of *ca.* 4 kb, which is present only in infected cells, was not pursued further as our interest was focused on the existence of an IE RNA. Conversely, however, this sensitive analysis indeed revealed an mRNA species of >5 kb that became visible only after infection in the presence of CH, thus identifying it as an IE phase transcript. The blot did not reveal splice products that would be expected to be more stable and, thus, more prominent than a long splice precursor. In conclusion, m164 sequence is indeed present in distinct mRNA species of *ca.* 2 kb and >5 kb expressed in the E and the IE phase, respectively.

### 2.3. Mapping of the m164 E Phase Start Sites of Transcription and Translation

It was obvious to predict that the *ca.* 2 kb mRNA that is abundantly expressed in the E phase codes for the previously identified E phase protein m164/gp36.5 [22], which is considerably smaller than the gp48.6 (includes 2 kDa from the identified single *N*-glycosylation [22]) predicted for a protein starting at the first of three AUG (Met1, Met27, and Met91) translation start sites in the predicted ORF*m164* [33,34]. In fact, relative to Met1, the *N*-terminus of the mature protein was definitively identified by mass spectrometric sequence analysis to be Ser108. We, therefore, originally speculated that the translation start site might be the third AUG (Met91). Accordingly, we proposed a 17-aa signal peptide of which existence was bioinformatically supported by the SignalP 3.0 algorithm [35] predicting signal peptide peptidase cleavage between Ala107 and Ser108 [22].

**Figure 3:**
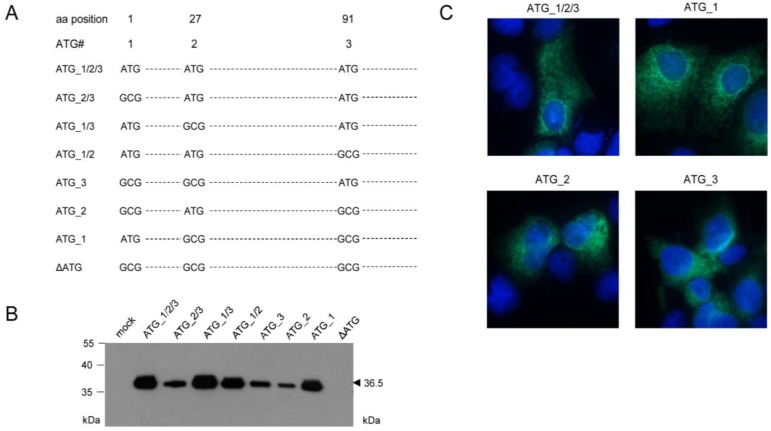
Mapping of AUG start codon usage by comprehensive mutational analysis through cell transfection with a complete set of expression plasmids. (**A**) Overview scheme of combinatorial ATG to GCG mutations for the three ATG codons present in the predicted ORF*m164*; (**B**) Western blot analysis of gene products of mutated *m164* ORFs. COS7 cells were transfected with 4 µg of expression plasmids carrying single or combined ATG to GCG mutations at potential start sites. After 48 h, total protein was extracted and 30 µg of the protein lysates were subjected to sodium dodecyl sulfate polyacrylamide gel electrophoresis (SDS-PAGE) (12.5%) followed by Western blot analysis for detection of m164 protein species by using polyclonal antibody directed against a *C*-terminal peptide; (**C**) m164/gp36.5 localization in the ER, independent of usage of the 1st, 2nd, or 3rd AUG as start codon. COS7 cells were seeded on glass coverslips and were transfected with 0.4 µg of pcDNA_m164_ATG_1/2/3, _1, _2, or _3. At 24 h after transfection, cells were fixed and protein m164/gp36.5 was detected with the polyclonal antibody. DNA was stained by Hoechst-dye.

With the initial aim to verify this assumption, mutational analysis was performed by transfection of COS7 cells with a complete set of expression plasmids comprising all possible combinations of ATG to GCG mutations of the three proposed start codons (Figure 3A). This analysis revealed that all three AUG start codons can be used, at least upon transfection of COS7 cells, when usage is enforced by absence of competing start codons. Unexpectedly, however, the different-lengths primary translation products were all co- or posttranslationally processed to yield the 36.5 kDa mature protein (Figure 3B), which also showed the typical ER and nuclear rim/outer nuclear membrane localization (Figure 3C) described for protein m164/gp36.5 previously [22]. Translation was prevented only by mutation of all three ATG in expression plasmid ΔATG (Figure 3B), thus showing that no start codon other than AUG is used.

**Figure 4:**
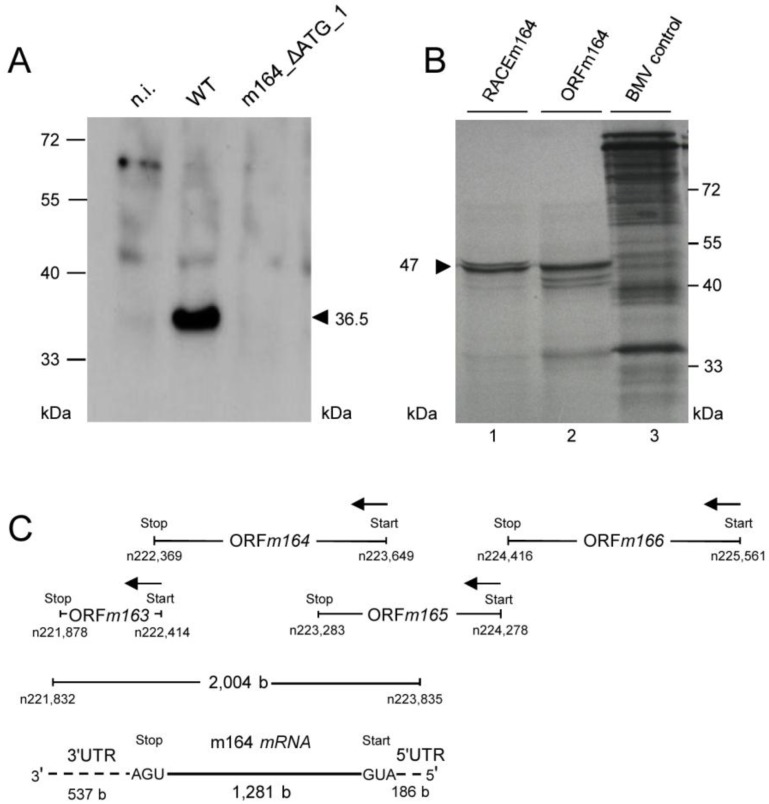
(**A**) Verification of the 1st AUG as start codon. MEF were infected with mCMV-WT.BAC or mCMV-m164_ΔATG_1, and at 6 h p.i. proteins were extracted and 30 µg of whole protein lysates were subjected to SDS-PAGE (12.5%) followed by Western blot analysis detecting protein m164/gp36.5; (**B**) Verification of the E phase mRNA encoding the ORF*m164* product. The *in vitro* transcribed RNAs (see Appendix Figure A1) were used as templates for *in vitro* translation. Shown is the autoradiograph of [^35^S]-labeled translation products separated by SDS-PAGE. Lane 1: proteins translated from the RACE*m164 in vitro* transcript. Lane 2: proteins translated from the ORF*m164 in vitro* transcript. Lane 3: BMV (Brome Mosaic Virus) RNA control supplied in the Ambion MEGAscript SP6 kit (Life Technologies, Catalog No. AM1330, Darmstadt, Germany); (C) Map of the m164 mRNA and its localization as revealed by 5'- and 3'-RACE shown in Appendix Figure A1.

This analysis, however, did not reveal the start AUG that is actually used when any of the three can be chosen, although increased signal strengths with expression plasmids containing ATG_1, alone or in combinations, indicated preferential usage of the first AUG (Figure 3B). Translation start at the first AUG is also strongly suggested by ribosome profiling, showing reads beginning at position -12 relative to the AUG, which is the correct distance from a start site [36,37]. To finally settle this question under the more real conditions of infection, we constructed recombinant virus mCMV-m164_ΔATG_1, with only the first start codon mutated, to infect MEF. As revealed by Western blot analysis, this mutation abolished expression of gp36.5 (Figure 4A).

**Appendix Figure A1:**
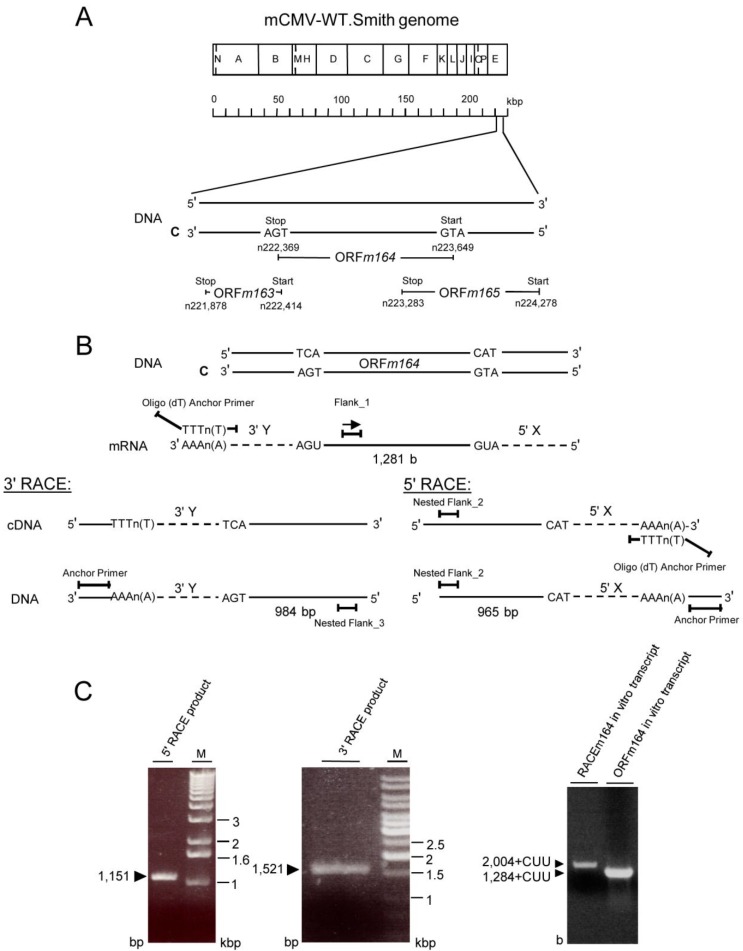
Mapping of the E phase m164 transcript with its 5' and 3' untranslated regions (UTRs). (**A**) *Hin*d III restriction map [61] of the mCMV-WT.Smith genome (GenBank Accession No. MCU68299), and map position of the predicted ORF*m164* [34] located within the *Hin*d E fragment on the C (complementary) strand. It overlaps with neighboring ORFs *m163* and *m165* by 45 bp and 366 bp, respectively; (**B**) Schematic illustration of the strategy of 5' and 3' RACE for identifying UTRs. For 5' RACE, poly(A)^+^ mRNA is reverse transcribed using an internal primer Flank_1. An oligo-dA sequence is appended to the 3' end of the resulting m164 cDNA fragment. PCR performed with an oligo-dT anchor primer and an internal primer Nested Flank_2 will reveal the expected 965 bp of predicted ORF sequence (up to the complementary start codon CAT) plus an additional unknown number of 5' bp (5' X; dashed line). For 3' RACE, poly(A)^+^ RNA is reverse transcribed by using an oligo-dT anchor primer. The resulting cDNA is amplified by using the PCR anchor primer and an internal primer Nested Flank_3 to reveal the expected 984 bp of predicted ORF sequence (up to complementary stop codon TCA) plus an additional unknown number of 3' bp (3' Y; dashed line); (**C**) *Left two panels*: Ethidium bromide-stained 1% agarose gels identifying 1,151 bp 5' and 1,521 bp 3' RACE products, allowing the calculation of 5' X and 3' Y, respectively. Sequencing revealed 2,004 bp of non-overlapping sequence representing a 2,004 base-long mRNA, referred to as RACEm164 RNA. *Right panel*: *in vitro* transcripts. Ethidium bromide-stained agarose/formaldehyde gel showing the *in vitro* transcription products (1 µg RNA per lane) obtained with DNA templates representing either the full-length mRNA [except the poly(A) tail] as determined by RACE analysis (RACEm164) or the sequence of only the predicted ORF (ORF*m164*), both 5' extended with the 20-bp SP6 promoter sequence ATTTAGGTGACACTATAGAA. CUU, additional codon derived from the SP6 promoter sequence.

Having, thus confirmed ORF*m164*, the slightly more than 2 kb length of the predominant E phase mRNA exceeded the calculated 1281 b of the ORF, which is difficult to explain just by the poly(A)^+^ tail and rather suggests the existence of UTRs. To address this issue, rapid amplification of cDNA ends (RACE) analysis was performed and in fact identified 3' and 5' UTRs of 537 bp and 186 bp, respectively (Appendix Figure A1). This result defined a length of the mRNA of 2,004 bp (polyadenylated tail not included), which is compatible with the length revealed by Northern blot analysis of poly(A)^+^ E phase m164 transcripts (recall Figure 2). Finally, *in vitro* transcribed ORF RNA as well as RACE RNA (Appendix Figure A1C) were translated *in vitro* and both resulted in a dominant product of *ca.* 47 kDa (Figure 4B) in accordance with the calculated molecular mass of the unprocessed 46.6 kDa primary translation product starting at Met1 (see above). Thus, in conclusion, the *ca.* 2 kb E phase poly(A)^+^ RNA is, in fact, the one that codes for the E phase protein m164/gp36.5. To sum these results up, Figure 4C shows a revised map of m164 and its neighboring genes with which it largely overlaps.

### 2.4. Confirmation of an Upstream IE Phase RNA Encompassing the Complete m164 E Phase RNA Sequence

**Figure 5:**
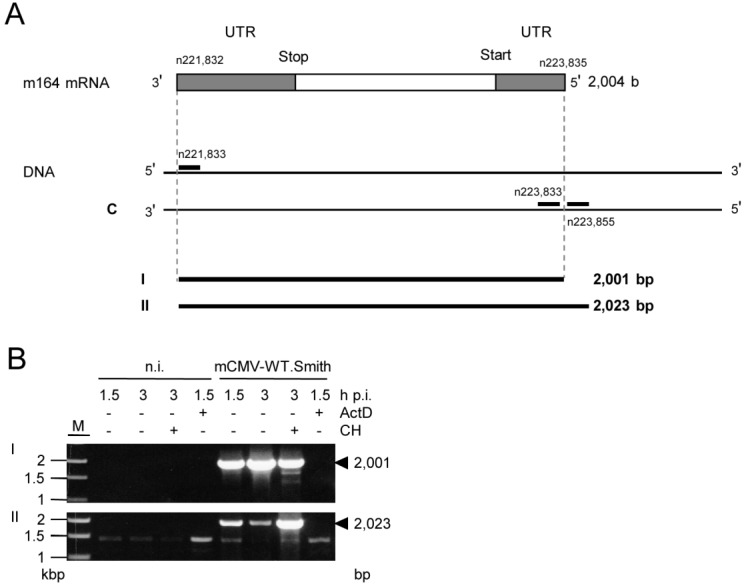
Verification of the m164 IE phase mRNA by RT-PCR. (**A**) Schematic map of the RACE-defined m164 mRNA with its 3'- and 5'-UTRs. RACE analysis (see Appendix Figure A1) predicted an mRNA of 2004 b. Positions of oligonucleotides binding inside or outside of the RACE product are indicated. Calculated sizes of the respective RT-PCR products are shown in lines I and II; (**B**) MEF were infected with mCMV-WT.Smith in presence (+) or absence (–) of ActD (5 µg/mL) or CH (100 µg/mL) as indicated. At 1.5 h or 3 h p.i. total RNA was isolated and subjected to RT-PCRs using oligonucleotides for product I or II (see Appendix Table A1). Amplificates were analyzed on a 1% agarose gel. M, marker; n.i. not infected; bp, basepairs. Controls with no RT step were negative throughout (not shown).

What, finally, is the >5 kb IE phase poly(A)^+^ RNA detected with the m164 sequence-specific riboprobe in the Northern blot (recall Figure 2)? RACE mapping of the complete m164 E phase transcript allowed us to design RT-PCRs with one primer placed at the 3' terminus and a second primer placed either to the left (RT-PCR I) or to the right (RT-PCR II) of the 5' terminus, that is inside or outside of the E phase transcript, respectively (for the strategy, see Figure 5A).

**Figure 6:**
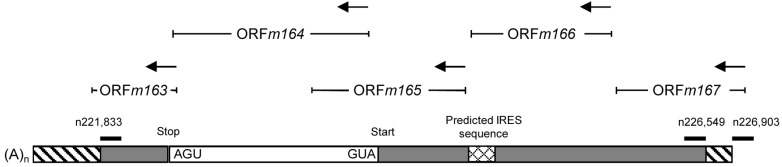
Provisional map of the >5 kb m164 IE mRNA and its localization within the mCMV genome. Positions of oligonucleotides binding inside or outside of the IE transcript are shown. Unmapped sequences are indicated as hatched boxes. A predicted IRES is indicated.

**Appendix Figure A2:**
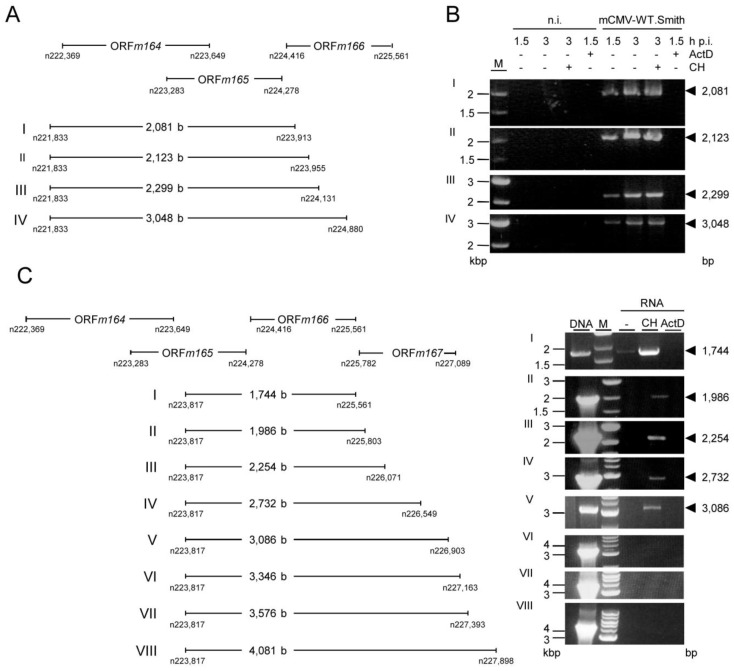
RT-PCR primer walking for searching the 5' end of the long IE m164 mRNA (**A**) Map indicating relative positions of ORFs *m164* to *m166*. I-IV show predicted lengths of RT-PCR products and the positions of the oligonucleotides on the mCMV genome (map not drawn to scale); (**B**) MEF were infected with mCMV-WT.Smith in presence (+) or absence (–) of ActD (5 µg/mL) or CH (100 µg/mL). At 1.5 h p.i. (for ActD) or 3 h p.i. (for CH), total RNA was isolated and subjected to RT-PCR. Products were analyzed on a 1% agarose gel stained with ethidium bromide. M, marker; n.i., not infected; (**C**) *Left panel*: map indicating relative positions of oligonucleotides and RT-PCR products (map not drawn to scale); *right panel*: MEF were infected with mCMV-WT.Smith in absence (–) or presence of CH or ActD, and total RNA was isolated at 3 h p.i. (for CH) or 1.5 h p.i. (for Act.D) and subjected to RT-PCR using oligonucleotides for products I-VIII (see Appendix Table A1). DNA isolated from infected cells not treated with inhibitors was used as a positive control.

With total RNA from infected cells, the inside 5'-end primer resulted in the expected amplificate of 2,001 bp in RT-PCR I, corresponding to the RACE-mapped E phase mRNA. Notably, in RT-PCR II, the outside 5'-end primer also gave an amplificate, thus indicating the existence of an RNA that initiates upstream of the 5' end and includes the complete m164 E phase transcript sequence (Figure 5B). As RT-PCR I detects both transcripts, the signal was only in part sensitive to CH treatment of the infected cells, with the remaining signal coming from IE RNA. In contrast, an enhanced signal seen by RT-PCR II after CH treatment shows that it results from IE RNA. Upstream “walking” of the 5'-end primer (Appendix Figure A2) located the start site of this IE mRNA between nucleotides n226,549 and n226,903 within ORF*m167* (for a summarizing map, see Figure 6), resulting in a length of >5 kb compatible with the length seen for the IE phase poly(A)^+^ RNA detected by the Northern blot analysis (recall Figure 2). As this RNA ends before the start of the predicted ORF*m167*, it cannot be the regular m167 transcript, whereas ORF*m166* [38] is completely included. Notably, in accordance with IE gene expression upstream of *m164*, by using an mCMV ORF microarray, the group of T.E. Shenk has defined a cluster of IE genes within restriction fragment *Hin*d IIIE, namely genes *m166-m169* [39,40,41].

As the full-length m166 mRNA with a putative 5' UTR has not yet been mapped, it might overlap with ORF*m167* so that the transcription start site of m166 mRNA remains a candidate. Alternatively, the long m164 epitope sequence-encompassing IE phase mRNA might represent a new transcript using an alternative start site within ORF*m167*. Theoretically, as no in-frame translation start sites are present upstream of ORF*m164*, the IE mRNA might represent a polycistronic transcript using the ORF*m164* start site, either directly or with an upstream internal ribosomal entry site (IRES). Bioinformatical analysis [42] indeed revealed a putative group 4 IRES [43] 1,044 bp upstream of the confirmed m164 AUG. Though IRES sequences are rarely described for herpesviral transcripts, it remains to be tested if this IRES is functional.

As Western blot analysis with antibodies directed against a *C*-terminal peptide of ORF*m164* did neither detect m164/gp36.5 nor larger or smaller isoforms expressed in the IE phase ([22], and data not shown), translation from the long IE mRNA and consequent m164 epitope presentation in the IE phase may result from a smaller isoform or even oligopeptide lacking the *C*-terminus.

In any case, this study has revealed the existence of an IE phase mRNA that starts upstream of the E phase m164 transcript and that spans the complete m164 sequence, including the sequence that encodes the CD8 T-cell epitope of which presentation was, so far, assigned only to the E phase.

## 3. Experimental Section

### 3.1. Cells, Viruses, and Mice

Primary BALB/c or C57BL/6 mouse embryo fibroblasts (MEF) were prepared as described previously [44] and cultivated in modified Eagle’s medium (MEM) supplemented with 10% fetal calf serum (FCS) and antibiotics. For transfection experiments, COS7 cells were cultivated and seeded 24 h prior to transfection in DMEM medium supplemented with 10% FCS and antibiotics.

The m164 peptide (AGPPRYSRI)-specific, polyclonal cytolytic T-lymphocyte (CTL) line (m164-CTLL), and the SIINFEKL-specific CTLL were generated and tested for functional avidity as described previously [44,45].

High virus titer stocks of mCMV Smith strain (mCMV-WT.Smith; ATCC VR-194/1981 re-accessioned as VR-1399), mCMV.WT.BAC [46], mCMV-m164Ala [13], mCMV-SIINFEKL [29], and mCMV-SIINFEKA [29] were prepared from infected MEF according to standard procedures [44,45]. Unless stated otherwise, infection was regularly performed with mCMV-WT.Smith.

BALB/c and C57BL/6 mice were bred and maintained under SPF conditions at the “Central Laboratory Animal Facility (CLAF)” of the University Medical Center Mainz. All experimental procedures were performed in compliance with the “International Guiding Principles for Biomedical Research Involving Animals” guidelines [47]. The experiments were approved according to German federal law under permission number AZ 23 177-07/G 09-1-004.

### 3.2. Infection Conditions

Confluent MEF were infected in the second or third cell culture passage under conditions of centrifugal enhancement of infectivity with 0.2 PFU/cell of the indicated viruses, resulting in an effective multiplicity of infection (MOI) of ~4 PFU per cell. For viral transcriptional arrest in the IE-phase, cells were infected in presence of cycloheximide (CH; 100 µg/mL; Sigma-Aldrich, Catalog No. C7698, Steinheim, Germany) and, 3 h later, CH was replaced with Actinomycin D (ActD; 5 µg/mL; Sigma-Aldrich, Catalog No. A9415, Steinheim, Germany). For E phase arrest, cells were infected in the presence of phosphonoacetic acid (PAA; 250 µg/mL, Catalog No. 284270, Steinheim, Germany) [44].

### 3.3. Construction of Expression Plasmids

To amplify the full-length sequence of ORF*m164*, PCR was performed using mCMV-WT.BAC genomic DNA as template with oligonucleotides m164_rev_BamHI and m164_full_*Hin*d III (Appendix Table A1) with the following protocol parameters: 5 min at 95 °C; 35 cycles of 10 s at 94 °C, 30 s at 60 °C, and 40 s at 72 °C followed by final primer extension for 10 min at 72 °C. The PCR product was subcloned into vector pcDNA3.1 after *Bam*H I/*Hin*d III restriction. To insert nucleotide exchanges ATG to GCG at nt positions 223, 649–223, 647; 223, 571–223, 569 and 223, 379–223, 377 the construct was subjected to site-directed mutagenesis using the Quick Change II Site-Directed Mutagenesis Kit (Agilent, Catalog No. 200524, Böblingen, Germany) with the oligonucleotide pairs m164_1stATG_for/rev, m164_2ndATG_for/rev, and m164_3rdATG_for/rev (Appendix Table A1), respectively. The mutagenesis resulted in constructs pcDNA-m164_ATG_2/3, pcDNA-m164_ATG_1/3, and pcDNA-m164_ATG_1/2. Plasmid pcDNA-m164_ATG_2/3 was subjected to a second round of mutagenesis using the respective oligonucleotides resulting in the constructs pcDNA-m164_ATG_3 and pcDNA-m164_ATG_2. Plasmid pcDNA-m164_ATG_1/3 was used as template for construction of pcDNA-m164_ATG_1. A third round of mutagenesis using pcDNA-m164_ATG_3 as a template resulted in pcDNA-m164_ΔATG. The successful replacement was confirmed by sequencing (GATC; Konstanz, Germany).

### 3.4. Construction of Recombinant mCMV

To generate recombinant virus mCMV-m164_ΔATG_1, BAC mutagenesis was performed by Red-mediated markerless DNA recombination as described by Tischer and colleagues [47]. In brief, oligonucleotides pEPKan-S_m164_1stATG__for and pEPKan-S_rev (see Appendix Table A1) were used for a PCR with plasmid pEP-kanS [48] as a template. The resulting products were subjected to a second round of PCR using oligonucleotides pEPKan-S_m164_1stATG_rev and m164_1stATG_short. The resulting product was transformed into GS1783 cells carrying WT.BAC. After Red-recombination, arabinose-induced I-*Sce*I expression, and a second round of Red-recombination, m164_ΔATG_1 BAC DNA was purified, and successful replacement of the ATG was confirmed by sequencing (GATC; Konstanz, Germany).

### 3.5. Protein Extraction and Analysis

MEF were seeded on 10-cm diameter cell culture dishes and infected with an MOI of 4 under conditions of centrifugal enhancement of infectivity. At defined times p.i., cells were washed with ice-cold PBS and scraped-off. After centrifugation (5 min, 3,000 rpm, 4 °C), the pelleted cells were lysed for 15 min on ice in 200 µL Lysis Buffer/dish composed of 0.2 M NaCl, 1.5 mM MgCl, 4 mM EDTA, 4 mM EGTA, 1% Triton X-100, 20 mM HEPES; with complete proteinase inhibitor (diluted 1:25; Roche, Catalog No. 11697498001, Mannheim, Germany) and 1 mM DTT added shortly before use. After centrifugation (10 min, 14,000 rpm, 4 °C), supernatants were collected and the amount of protein was determined by BCA-Assay (Thermo Scientific, Catalog No. 23225, Dreieich, Germany). An amount of 30 µg of total protein was separated on an SDS-PAGE followed by Western blot analysis. Detection of m164 proteins was performed with polyclonal affinity-purified rabbit antibodies directed against a *C*-terminal peptide (1:500) [49] and an HRP-conjugated anti-rabbit antibody as secondary Ab (DakoCytomation, Catalog No. P045001, Hamburg, Germany).

### 3.6. Transfection

A total of 5 × 10^5^ COS7 cells per 10-cm diameter dish were seeded and 4 µg DNA was transfected with Polyfect (Qiagen, Catalog No. 301105, Hilden, Germany) according to the manufacturer’s instructions. Forty-eight hours later, cells were harvested and total protein was extracted as described above. An amount of 30 µg of protein lysates were separated on a 12.5% SDS-PAGE followed by m164 protein-specific Western blot analysis.

### 3.7. Immunofluorescence Analysis

COS7 cells seeded on glass coverslips and transfected with 0.4 µg DNA were fixed with 4% (wt/vol) paraformaldehyde in PBS supplemented with 4% (wt/vol) sucrose. After incubation for 1 h in blocking buffer (PBS with 0.3% (vol/vol) Triton X-100 and 15% (vol/vol) FCS), intracellular m164 protein was detected by polyclonal affinity-purified rabbit antibodies (1:100) [49] and Alexa Fluor 546-conjugated goat anti-rabbit antibody (Life Technologies, Catalog No. A11010, Darmstadt, Germany) as secondary antibody. Cell nuclei were stained by 5 min incubation with the DNA-binding blue fluorescent dye Hoechst 33342 (1:5,000; Life Technologies, Catalog No. H3570, Darmstadt, Germany).

### 3.8. Northern Blot using RNA Probes

MEF were treated 15 min before infection either with 100 µg/mL CH or 5 µg/mL ActD, or were left untreated. Cells were infected under conditions of centrifugal enhancement. Highly purified polyadenylated RNA was isolated from the cell lysate at 1.5 h (untreated and ActD treated cells) or 3 h (untreated and CH treated cells) p.i. by using oligo(dT)-coated superparamagnetic 50-nm diameter microbeads (µMACS mRNA isolation kit; Miltenyi Biotec Systems, Catalog No. 130-075-201, Bergisch-Gladbach, Germany). To generate two complementary hybridization probes, a 431 bp DNA fragment of the m164 sequence (nt 222,625–nt 223,055) was amplified by PCR using the primers mRNAint_for and mRNAint_rev and the plasmid pSM3fr [46] as template. The resulting PCR product was subcloned into pDrive cloning vector (Qiagen, Catalog No. 223122, Hilden, Germany) resulting in pDrive-m164probe. After linearization of the plasmid with BamHI for transcription with T7, the MAXIscript Kit SP6/T7 (Life technologies, Catalog No. AM1320, Darmstadt, Germany) was used to synthesize single strand RNA probes labeled by random-priming with the DIG RNA Labeling Mix (Roche, Catalog No. 1277073, Mannheim, Germany) in the presence of DIG-UTP:dTTP (1:3). Amounts of 4 µg of poly(A)^+^ RNA or 2 µg of total RNA per lane were separated in 1.2% Agarose/37% formaldehyde gels and transferred onto positively charged nylon membranes. An amount of 700 ng of the DIG-labeled RNA-probe (T7) was diluted in 70 mL DIG Easy Hyb buffer prior to hybridization, which was performed as described above.

### 3.9. Mapping of mRNA by 5'/3'RACE

To map the full-length m164 transcript by 5'/3' RACE (rapid amplification of cDNA ends) [50], the 2nd generation kit (Roche, Catalog No. 03353621, Mannheim, Germany) was used for the analysis of poly(A)^+^ RNA isolated in the E phase of the viral replicative cycle at 6 h post-infection (p.i.).

In brief, poly(A)^+^ RNA was enriched from total RNA by the Oligotex mRNA Mini Kit (Qiagen, Catalog No. 72022; Hilden, Germany) according to the manufacturer’s instructions. 5' RACE analysis started with an RT-step transcribing m164 mRNA partially into cDNA by using the Flank_1 primer (see Appendix Table A1), followed by digestion of residual poly(A)^+^ RNA with RNase H. The transcription start site of gene m164 was identified by tailing the cDNA at its 3' end using terminal deoxynucleotidyl transferase with substrate dATP. The 3' region of the cDNA [corresponding to the 5' end of the poly(A)^+^ RNA] was then amplified using a standard PCR protocol with the provided oligo-dT anchor primer and the antisense primer Nested_Flank_2 (see Appendix Table A1) designed to bind internally within the predicted ORF*m164*.

3' RACE analysis started with the synthesis of cDNA using the oligo-dT anchor primer, followed by digestion of residual poly(A)^+^ RNA with RNase H. The 5' region of the cDNA (corresponding to the 3' end of the poly(A)^+^ RNA) was then amplified by using the provided PCR anchor primer and the ORF*m164* sequence-specific internal primer Nested_Flank_3 (see Appendix Table A1). Reaction products were analyzed by 1.5% agarose gel electrophoresis and were sequenced.

### 3.10. Primer Walking

Fifteen minutes prior to infection, MEF were either treated with 100 µg/mL CH or 5 µg/mL ActD, or were left untreated. Infection was performed under conditions of centrifugal enhancement. Highly purified polyadenylated RNA was isolated from the cell lysate at 1.5 h (untreated and ActD treated cells) or at 3 h (untreated and CH treated cells) p.i. by using oligo(dT)-coated superparamagnetic 50 nm-diameter microbeads (µMACS mRNA isolation kit; Miltenyi Biotec Systems, Catalog No. 130-075-201, Bergisch-Gladbach, Germany). For reverse transcription reactions, the OneStep RT-PCR Kit (Qiagen, Catalog No. 210210, Hilden, Germany) was used according to the manufacturer’s protocol. Reactions were carried out with an automated thermal cycler (GeneAmp PCR System 9700; Life technologies, Darmstadt, Germany), using the primer pairs shown in Appendix Table A1. The time-temperature profiles were as follows: reverse transcription for 30 min at 50 °C, initial PCR activation step for 15 min at 95 °C, 35 cycles of denaturation for 30 s at 94 °C, and annealing for 30 s with annealing temperatures and elongation conditions specified in Appendix Table A2. Final extension was performed for 10 min at 68 °C or 72 °C, dependent on the respective elongation temperature. Amplification products were visualized by standard procedures of 1% (wt/vol) agarose gel electrophoresis with ethidium bromide.

### 3.11. *In Vitro* Transcription and Translation

To introduce the SP6 promoter sequence [51] in front of the RACEm164 and the ORF*m164* sequence, PCRs were performed with primer pairs SP6_m164RACE_for and SP6_m164RACE_rev or SP6_m164*ORF*_for and SP6_m164*ORF*_rev (see Appendix Table A1), respectively. An amount of 1 µg of purified PCR product was used as template for *in vitro* transcription according to the manufacturer’s protocol for “Transcription Reaction Assembly” of the Ambion MEGAscript SP6 kit (Life technologies, Catalog No. AM1330, Darmstadt, Germany). After 3.5 h of incubation at 37 °C for transcription, DNA template was removed by digestion with DNase, and the RNA was cleaned with RNeasy Mini Elute Cleaning kit (Qiagen, Catalog No. 74134, Hilden, Germany) and analyzed on a 1% agarose/formaldehyde gel.

*In vitro* translation of the *in vitro* transcripts was performed with the Promega wheat germ extract (Promega, Catalog No. L4380, Mannheim, Germany). The reaction conditions were 90 min at 25 °C with 80 ng of template RNA. The resulting protein was labeled with Redivue L-[^35^S] methionine (Amersham Biosciences, Catalog No. AG1094, Little Chalfont, UK) and analyzed by 12.5% SDS-PAGE followed by autoradiography.

### 3.12. ELISpot Assay

Presentation of the m164- and SIINFEKL-epitope was determined after endogenous antigen processing in infected MEF (BALB/c, haplotype H-2^d^ and C57BL/6, haplotype H-2^b^, respectively) by using cognate CTLL as responder cells in a standard IFN-γ-based ELISpot assay. For selective arrest of the viral gene expression in the IE or E phase, MEFs were treated with inhibitors as described previously [44]. The assay was performed as described ([52,53] and references therein) and frequencies of IFN-γ-secreting cells and the corresponding 95% confidence intervals were calculated by intercept-free linear regression analysis using the software Mathematica [54].

## 4. Conclusions and Outlook

These data have revealed a paradigmatic example of a CD8 T-cell epitope that is encoded by two overlapping mRNAs expressed in different phases of the coordinately regulated gene expression program in the productive viral cycle [55,56]. As overlapping genes are a more general feature of CMV genomes [33,57], more such cases might be found in the future and lead to a revision of the assignment of antigenic peptides to a certain kinetic class in epitope listings. This is of relevance for the order in which epitopes are expressed relative to immune evasion proteins or for discussing viral gene expression during CMV latency. In the specific case of the memory inflation-inducing m164 peptide of mCMV it was assumed that the expansion of the m164 epitope-specific memory CD8 T-cell pool indicates E gene expression during latency [11,20,21]. With the new knowledge presented here, future experiments will have to address the question of whether the m164 peptide that is supposed to drive m164-specific memory inflation during latency indeed results from the E phase transcript or, alternatively, results from the here identified IE phase transcript. Sporadic expression of IE genes from the major IE locus during viral latency has been documented [27,58,59,60]. Thus, “noncanonical” expression and presentation of the m164 epitope from the long IE phase transcript could explain m164-specific memory inflation during viral latency in absence of reactivation of the coordinately regulated viral transcriptional program.

## Appendices

**Appendix Table A1:** Oligonucleotides used in this study; nucleotides inserting ATG to GCG substitutions are highlighted in bold face red letters.

Name	Sequence
pEPKan-S_m164_1stATG_for	5'-CCTGCCAGATCTTGGGGCCTCGGGTCGCCACACACGTGGGAAGGCGTTTCTCCGCGGCTGTCGGCAACCAATTAACCAATTCTGATTAG-3'
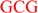
pEPKan-S_m164_1stATG_rev	5'-GCTGCGCGTCTCCCCCTCCGACAGCCGCGGAGAAACGCCTTCCCACGTGTGTGGCGAGGATGACGACGATAAGTAGGG-3'
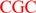
m164_1stATG_short	5'-CCTGCCAGATCTTGGGGCCTCGGG-3'
pEPKan-S_rev	5'-AGGATGACGACGATAAGTAGGG-3'
mRNAint_for	5'-GGCTTCTCGGGCCTCGTCGTGTGC-3'
mRNAint_rev	5'-GCCACGTGGACGGGTCGACATTCG-3'
m164mRNA_rev	5'-ACGCACATCACCAGTG-3'
mRNAin_for	5'-GTTTAGTCGGGAGCGTG-3'
m164mRNAout_for	5'-GTGAGGTGGTGGATAGATAC-3'
m164mRNAout50_for	5'-GTGTGAGCAGTATCTGGG-3'
m164mRNAout100_for	5'-GAGACGGCTAGCGAATC-3'
m164mRNAout300_for	5'-GCGGACTACATCTCGATC-3'
m164mRNAout1000_for	5'-GAGTGGAGCGAGAACAAC-3'
m164mRNAin_rev	5'-CACGCTCCCGACTAAAC-3'
m166_ORF_end_for	5'-ATGGCGCTTCCAGCAATTCC-3'
m166_ORF_end250_for	5'-CAGGTTCCGCAACAGTC-3'
m166_ORF_end500_for	5'-ATCGGATGGTGCTTCAG-3'
m166_ORF_end1000_for	5'-AAGCCTCCGAGGAAGAC-3'
m166.5_ORF_end_for	5'-GGCGGTAGAACAGTTGC-3'
m166.5_ORF_end250_for	5'-CCTTTGAGTGTGGAAAGTTTG-3'
m166.5_ORF_end500_for	5'-TTACCCGCGTCACATGG-3'
m166.5_ORF_end1000_for	5'-CCGTCCTTAGGTTTCGTTTC-3'
Flank_1	5'-CACGAAGGCGGCGAGGAAAC-3'
Nested Flank_2	5'-GACGACGCACGATACGCAGAC-3'
Nested Flank_3	5'-CTCATCTACGCGGCGGCTTCAG-3'
m164_rev_BamHI	5'-ATATGGATCCGGAGAAGAGTCAGAATCATGACGAACG-3'
m164_full_HindIII	5'-ATATAAGCTTCTCCTGCCAGATCTTG-3'
m164_3rdATG_rev	5'-GCGTAGATGAGGGCGAGCCCGGCCTTGAAGAGTCG-3'
m164_3rdATG_for	5'-CGACTCTTCAAGGCCGGGCTCGCCCTCATCTACGC-3'
m164_2ndATG_rev	5'-GCAACGCGTCGTCGGCACGGCAACTCGGGTTCCCCG-3'
m164_2ndATG_for	5'-CGGGGAACCCGAGTTGCCGTGCCGACGACGCGTTGC-3'
m164_1stATG_rev	5'-CCGACAGCCGCGGAGAAACGCCTTCCCACGTGTGTG-3'G
m164_1stATG_for	5'-CCACACACGTGGGAAGGCGGCGTTTCTCCGCGGCTGTCG-3'

**Appendix Table A2:** PCR cycling conditions used in this study.

Product	Primer rev	Primer for	Annealing	Elongation	Size [bp]
Figure 4					
I	m164mRNA_rev	m164mRNAin_for	51.7 °C	1 min 30 s; 72 °C	2,001
II	m164mRNA_rev	m164mRNAout_for	51.7 °C	90 s; 72 °C	2,023
Figure A2A					
I	m164mRNA_rev	m164mRNAout50_for	51.7 °C	2 min10 s; 68 °C	2,081
II	m164mRNA_rev	m164mRNAout100_for	51.7 °C	2 min10 s; 68 °C	2,123
III	m164mRNA_rev	m164mRNAout300_for	51.7 °C	2 min 18 s; 68 °C	2,299
IV	m164mRNA_rev	m164mRNAout1000_for	51.7 °C	3 min; 68 °C	3,048
Figure A2C					
I	m164mRNAin_rev	m166_ORF_end_for	55 °C	1 min 30 s; 72 °C	1,744
II	m164mRNAin_rev	m166_ORF_end250_for	55 °C	1 min 30 s; 72 °C	1,986
III	m164mRNAin_rev	m166_ORF_end500_for	55 °C	2 min 18 s; 68 °C	2,254
IV	m164mRNAin_rev	m166_ORF_end1000_for	55 °C	3 min; 68 °C	2,732
V	m164mRNAin_rev	m166.5_ORF_end_for	55 °C	3 min; 68 °C	3,086
VI	m164mRNAin_rev	m166.5_ORF_end250_for	55 °C	3 min 20 s; 68 °C	3,346
VII	m164mRNAin_rev	m166.5_ORF_end500_for	55 °C	3 min 30 s; 68 °C	3,576
VIII	m164mRNAin_rev	m166.5_ORF_end1000_for	55 °C	4 min; 68 °C	4,081
